# Federated and reusable processing of Earth observation data

**DOI:** 10.1038/s41597-025-04513-y

**Published:** 2025-02-01

**Authors:** Matthias Mohr, Edzer Pebesma, Jeroen Dries, Stefaan Lippens, Bram Janssen, Daniel Thiex, Grega Milcinski, Benjamin Schumacher, Christian Briese, Michele Claus, Alexander Jacob, Paulo Sacramento, Patrick Griffiths

**Affiliations:** 1 University of Münster, Institute for Geoinformatics, Münster, Germany; 2 VITO NV, Mol, Belgium; 3 Sinergise, Ljubljana, Slovenia; 4 EODC GmbH, Vienna, Austria; 5 Eurac Research, Bolzano, Italy; 6 Solenix Engineering Italia, Frascati, Italy; 7 European Space Agency, Earth Observation Programmes, ESRIN, Frascati, Italy

**Keywords:** Environmental impact, Technology

## Abstract

The unprecedented and continuously growing volume of Earth Observation (EO) and geospatial data has necessitated a paradigm change where compute is collocated with the data archives in public clouds. However, as no single cloud platform can host all of this data, federated processing solutions that work across multiple cloud platforms are becoming increasingly relevant. A community-based approach to federated processing has started using openEO, a common Application Programming Interface (API) and set of well-defined processes that simplifies reuse and provides a valuable level of abstraction when handling large EO data volumes. We present key concepts for federated processing and related interoperability aspects based on *openEO Platform*, a federated public cloud platform.

## Introduction

Earth Observation (EO) data available to users today is unprecedented in terms of quality, (spatial, temporal, radiometric) resolution and quantity. EO and geospatial data have become an integral component for science to address key societal challenges such as the climate or sustainability crises. Over the last two decades, key EO data archives have become freely accessible. Current and emerging EO programmes, such as the European Union Copernicus programme, are acquiring large data volumes that are made publicly available. With the continuously growing EO data archives, also the challenges of handling these data volumes efficiently have increased.

As EO data volumes started increasing, many academic and research institutions built their own cloud-like infrastructures, where they downloaded data and processed it locally. As such infrastructures were typically built in an ad-hoc and incremental fashion, using different technology stacks, this has in turn led to a very limited sharing of code involving data analysis, processing algorithms, and workflows.

Compared to other scientific disciplines that handle large data amounts such as bioinformatics^1^ and high-energy physics^2^, EO data combines the characteristics that it

- consists of a mix of classified, commercial and openly available datasets,
- has been collected by a limited set of sensors, operated by governmental or inter-governmental agencies, or by private companies, and
- concerns observations of the dynamics of a single object (Earth) under uncontrolled conditions.

The fields of meteorology and climate modelling also generate large Earth-related datasets (e.g. ECMWF Reanalysis v5 (ERA5)^3^, and Coupled Model Intercomparison Project Phase 6 (CMIP6)^4^). Compared to these two fields, EO data is acquired through observation rather than modelling, is often of higher temporal and spatial resolution, and does not involve forecasting. It requires modelling to obtain the variable of interest from the observed variables. These characteristics contribute to the way EO data has been analysed in the past, how it is analysed currently and how the field is changing.

As the data volumes became larger, fewer institutions could keep up with replicating data archives locally. It is now widely accepted that, if anywhere, public clouds are the only places that have the capacity to store and provide access to complete EO data archives. Consequently, some of the major space agencies as providers of these data are moving their processing systems and archives into public clouds. Today, global EO data archives have reached sizes of tens to hundreds of petabytes^5^.

EO data is commonly processed by the data providers from “raw” satellite observations to increasingly higher processing levels. For a number of sensors, these are stored as raster data, typically with one file for each spectral band or sensor mode. The acquired data is arranged in so called *tiles* (or *scenes*, or *granules*) within a given coordinate reference system (i.e., map projection). For example, a Sentinel-2 tile covers a fixed 100 km by 100 km region within the Universal Transverse Mercator (UTM) projection and contains image data for all 13 spectral bands in individual files. Due to the UTM grid layout, adjacent tiles overlap by about 10 km. When users intend to work over larger areas and/or long time series, the number of tiles that need to be processed can become very large, rendering the conventional local downloading approach highly inefficient. Moreover, processing such data in cloud computing environments typically involves first identifying all tiles concerned, and then iterating over them during processing and analysis. Finally, these datasets are often stored in compressed form and require decompression prior to analysis. As a consequence of all these aspects, EO data users find themselves spending a large part of their time on setting up and maintaining data handling and management pipelines, often dealing with many intermediate files, leaving less time to spend on the actual research question or application context.

Low-level cloud computing approaches, such as those offered by the DIAS^6^ or Earth on AWS^7^, require users to rent virtual machines, log in, install software, identify the needed images on the cloud platform, and run the software. Although that approach is hard to beat for power and flexibility, it also requires system administration and software engineering skills that many end users do not have and are hesitant to acquire. On the other hand, high-level cloud computing approaches can provide valuable abstraction for data access and processing. Examples include platforms like Google Earth Engine^8^, Microsoft Planetary Computer^9^ or Sentinel Hub^10^. They employ the concept of *image collections*, which allow users to directly address a sensor- and processing-level specific data archive, such as, for example, Sentinel-2 L2A. The file-based perspective, with individual tiles and file formats, is abstracted from the user and the entire archive is accessible by specifying a spatial and temporal extent. A related data model commonly provided to users of high-level cloud computing platforms is that of a *data cube*. Data cubes are multi-dimensional arrays with pixel level observations aligned temporally and spatially, which greatly simplify subsequent analysis. Data cubes are conventionally based on chunking and physical replication of the source data. Virtual data cubes, on the other hand, provide a view relative to the stored source data and the actual data cube is created lazily, on the fly, once processing is initiated^11^.

The high-level cloud platforms mentioned above have a number of potential limitations: the datasets available may be limited and not under the control of users, the software used may be specific to that platform and have limited possibilities of being extended by the user, and the user may have limited control of the cost management or compute resource allocation. Another common consequence is vendor lock-in: after investing considerable effort in using a platform, it becomes very costly to move analysis workflows to a different platform with its specific frameworks, conventions, and syntax. In many cases, it is not possible to reuse code that was developed within one platform on another platform. This also impacts scientific replicability: although it is theoretically possible to carry out cross-platform validation by analysing the same data on two platforms that provide the required computational processes, in practice this is never done because of the effort it takes, the limited scientific reward for it, and the lack of a community willing to discuss problems if they show up. This inability to independently reproduce EO data analysis processes across different platforms was one of the motivations for developing *openEO* (https://openeo.org).

*openEO*^12^ is a programming language agnostic, open Application Programming Interface that connects R, Python, JavaScript, and other clients to different cloud backends holding the large EO data archives, in a simple and unified way^13^. It provides a unique abstraction level that simplifies data management and enables an efficient way to reuse algorithms and workflows. Backends can be data centres that host data and processing infrastructures that allow to process data on a large scale.

It might be surprising to ascertain that not all Earth-related data is assumed to be provided on a single cloud platform, as infinitely scalable storage would make this possible. However, EO data archives of several petabytes are hard to move from one cloud platform to another and expensive to host. Another reason for them not being available on a single platform is their origin in disciplines different from Earth observation such as meteorology or climate modelling.

This paper focuses on *federated processing* of EO data in public clouds: analysing EO data that is not available within a single cloud platform. It discusses the technical challenges, including interoperability and reuse, associated with it and introduces *openEO Platform* (https://openeo.cloud) as an implementation. It is a public and operational platform that is built on top of the openEO specification and ecosystem. The platform federates a set of openEO-compliant cloud backends and offers multiple implementations of the openEO specification as a single independent service. Although openEO Platform is a specific federation, the concepts presented in this paper are not tied to any specific platform. The components are open-source and built around an open specification.

## Results

A community-based solution to federated processing of Earth Observation image collections is provided in *openEO Platform*. This platform implements the openEO API and processes. The API specification is available at https://api.openeo.org. The process specifications are available at https://processes.openeo.org, which includes all openEO processes mentioned in the following sections.

**Fig. 1:**
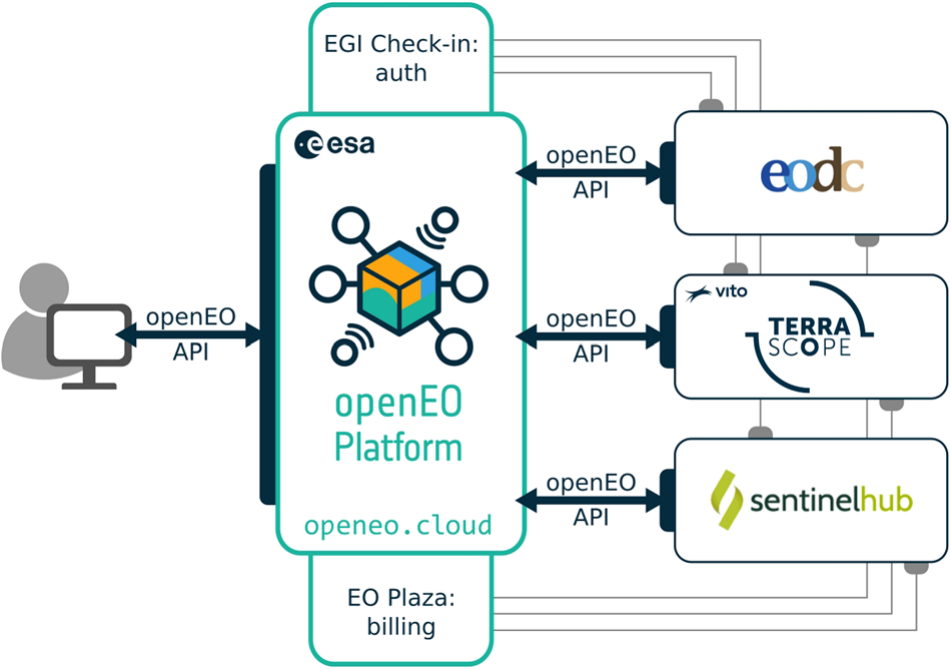
The federated components of openEO Platform. The end user, on the left, interacts with openEO Platform, via the so-called “aggregator” component in-between, through the standard openEO API protocol. Depending on the user’s request, openEO Platform interacts on behalf of the user with one or more openEO backends on the right, again using the standard openEO API protocol. Some additional components are shown here: authentication/authorisation aspects are handled by EGI Check-in using the standard OpenID Connect protocol and billing aspects are handled through a custom marketplace component.

*openEO Platform* is a collaborative effort of companies and research groups in Europe that bundles the capacity of several existing cloud platforms in a federated manner behind a single openEO API endpoint, https://openeo.cloud. Figure 1 visualises the high-level structure of the federation. An end user interacts with openEO Platform with the central federation components trough the openEO API protocol. These components are using the standard openEO API protocol again to interact with one or more backends.

In addition to the three backends (EODC, Terrascope, Sentinel-Hub) that are currently integrated into the *openEO Platform* there are a number of other openEO API implementations. They were mainly developed by companies and research groups to enable quick and easy in-house development (e.g., Open Data Cube by EURAC or the openEO GRASS GIS driver by mundialis) and range from proof of concepts (e.g., an openEO proxy for Google Earth Engine) to fully operational public offerings such as the openEO service within the Copernicus Data Space Ecosystem (CDSE). Due to their different nature and maturity, each backend offers different capabilities and features, but all follow the openEO API specification. The *openEO Hub* (https://hub.openeo.org/) provides an overview of openEO backend implementations and their capabilities.

The project that developed this platform was selected after a public procurement from the European Space Agency (ESA). The platform can be used by any user. Nevertheless it is a commercial service that must be self-sustainable and users can choose from three options (https://openeo.cloud/#plans). A *free trial version* is available for newly registered users. The *ESA Network of Resources (NoR)* offers ESA-sponsored usage of the platform for certain eligible entities. For all other entities, the NoR offers options to purchase the service offerings with a service-level agreement (SLA).

### Aggregator

The technical component that manages the incoming requests is called the *openEO Aggregator* (https://github.com/Open-EO/openeo-aggregator). It sits between the end user and the participating openEO data centres, handling multiple federation aspects:

- Merging and unifying general metadata listings, such as the union of data collections, processes, and file formats across the data centres. It also merges user-specific resources, such as batch job listings.
- Dispatching of simple processing requests to the appropriate data centres. Routing the processing requests is based on analysis of the availability of data and processes. Some minimal rewriting of requests or process graphs is required for internal housekeeping.
- Handling of complex processing requests that require multiple data centres. This is a more advanced feature, discussed in the next section, that involves automatic splitting of workflows, scheduling, process tracking, and merging results.

An important design choice for the implementation of the aggregator was to keep it lightweight and avoid assumptions on particular implementation details of participating backends. For example, the aggregator does not keep track of data processing tasks (batch jobs) itself, even if they are created through the aggregator. The aggregator fully depends on the participating backends to correctly list batch jobs and it just presents a merged listing to the end user.

The openEO API itself is defined in a way that a federation can be introduced without necessary changes to the implementations at the data centres (or client libraries). As they all implement the same API and workflow language, the implementations are interchangeable and the number of implementations is not limited.

Typically, users do not need to know which data centres are available and what they offer individually. Nevertheless, sometimes users want to select a specific data centre (or at least verify what was selected automatically) as they may have worked with it in the past or as it is closer to them. As such, an extension to the openEO API was developed, which is the *Federation Extension* (https://github.com/Open-EO/openeo-api/tree/master/extensions/federation). This extension adds information about the available backends and their status to the openEO API responses of the aggregator. It also adds information about the availability of data and processes for specific data centres to the responses, and details the data centres that have been involved in processing the results (lineage).

### Secure and efficient cross-backend processing

A federated processing component such as the openEO aggregator allows a direct execution of workflows that involve datasets (or processes) hosted across multiple data centres. Data processing workflows are called “(user-defined) processes” in openEO and consist of metadata and a process graph.

Two key aspects that need to be covered are direct communication between backends to exchange intermediate results without granting full user rights, and coordination of the dependencies between the processing steps that need to occur in multiple places. To address both aspects, the openEO aggregator component leverages the openEO process load_stac, which is a generic process to load data that has its metadata provided according to the SpatioTemporal Asset Catalog (STAC) specification (https://stacspec.org). In this particular case, it allows to import processing results from one backend to another, as openEO batch job results are exposed with STAC metadata by design. The openEO API also strongly recommends backends to provide a signed URL (Uniform Resource Locator) to point to processing results, which simplifies secure sharing between openEO backends, without the need for additional authentication and authorization measures. Moreover, the openEO API also supports “partial” batch job result listings, which makes it possible to pass around a batch job result URL that is valid even before the job has finished. This “partial” batch job result reference, provided as URL to load_stac, embodies the yet unfulfilled or fulfilled dependency between processing steps.

**Fig. 2:**
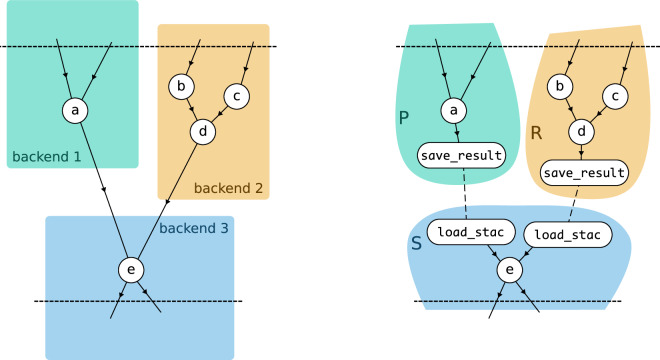
A cross-backend process graph splits where tasks span multiple backends. Nodes are assigned to backends, and dependencies crossing boundaries are replaced with save_result and load_stac nodes. This creates standalone subgraphs, each executable on its designated backend, ensuring efficient distributed processing.
  In practice, splitting of openEO process graphs is mainly steered by availability of EO data on different openEO backends. The data loading nodes of the process graph (load_collection nodes) are the starting point to figure out if and how the process graph can be split. There are often multiple ways and approaches to split a process graph (Fig. 3). The challenge is finding an optimal split, but there are multiple aspects that can be considered, which may also be prioritized differently: minimizing the number or volume of cross-backend data transfers, minimizing the overall run time or processing cost, maximizing certain aspects of caching, etc. Moreover, there are some unknowns (e.g., how large the intermediate data will be) or aspects with high variability (e.g., cluster load influencing the expected processing time). In an initial implementation, the aggregator was limited to just splitting off the data loading nodes (split points *A* or *B* in Fig. 3), which is the simplest option in terms of graph manipulation to unlock cross-backend processing in an openEO ecosystem. In a next step, the graph splitting was extended to search deeper into the graph to find better split points (like *C* or *D* in Fig. 3). For example, split point *C* from Fig. 3 comes after a reduce_dimension step, which typically implies a reduction of data (cube) size, and consequently smaller and faster data transfer, compared to split point *A*.

In more detail, the aggregator implementation solves cross-backend processing problem as follows:

1. The process graph is split into multiple subgraphs in such a way that each subgraph can be executed on a single backend. Edges from the original process graph that cross boundaries between data centres are replaced by a combination of a save_result process in one subgraph, to produce an intermediate result, and a corresponding load_stac process in another subgraph, to load that intermediate result. Figure 2 illustrates the cross-backend process graph splitting, focussing on where a process graph crosses the boundaries between backends. On the left, process graph node *a* must be processed on backend 1, nodes *b*, *c* and *d* are for backend 2, and *e*, which depends on the results of *a* and *d*, is for backend 3. On the right, the direct links *a* – *e* and *d – e* are replaced by combinations of new save_result and load_stac nodes, which effectively splits the original process graph in separate, stand-alone process graphs *P*, *R* and *S*, each of which can be executed on a different backend.

**Fig. 3:**
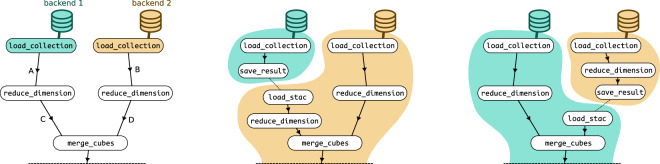
Illustration of different graph splitting strategies. Left: openEO process graph using two EO data sources, each loaded with a load_collection process, but one is only available on backend 1 and the other is only available on backend 2. Some more processing steps follow after that, such as merge_cubes, which merges its input data into a single data cube. This necessitates data transfer through cross-backend processing, and requires splitting the process graph in one of the four indicated split options *A*, *B*, *C* and *D* here. Middle: splitting the original process graph at split point *A* as discussed in Fig. 2 resulting in two new, stand-alone process graphs. Right: outcome of splitting at the split point *D*, which is deeper into the graph.
2. The subgraphs are submitted to the respective openEO backends, in an order such that a given subgraph (for example, *S* from Fig. 2), is submitted after the subgraphs it depends on (for example, *P* and *R* from Fig. 2). This is important because the signed URL of batch job results only becomes concrete after actually submitting the job to a backend. To make subgraph *S* concrete, with concrete load_stac nodes pointing to the results it depends on, the signed URLs of the results of *P* and *R* must be concrete first.
3. Although all subgraphs are submitted in a certain order, the aggregator submits them regardless of the status or availability of the results they depend on. For example, subgraph *S* from the example of Fig. 2 is submitted even if *P* or *R* are not finished yet. Because the usage of “partial” batch job result URLs in load_stac, the backends themselves can check when a dependency is available and start the actual processing. With this approach, the coordination responsibility is distributed across the openEO backends. The aggregator’s main involvement is in the initial phase where it has to take care of splitting the process graph and submitting the subgraphs accordingly. This avoids authentication and authorization challenges in an approach where the aggregator would actively have to monitor job states across backends and schedule jobs in the background on behalf of the user.

At this point, the end user is running a “virtual” cross-backend job at the level of the openEO aggregator, which is backed by several concrete “subjobs” that are running the relevant openEO backends in a fully distributed manner, with direct and secure communication between these backends.

### Harmonising the offering

For a consistent interface, some aspects of the offering of the data centres are harmonised in openEO Platform:

1. Specific data collections offered by multiple data centres and their STAC metadata
2. The set of core processes available in all data centres
3. Specific file formats and their parameters

These specific parts are intentionally not harmonised by the openEO API to allow as many use cases as possible through a generic specification, but for a consistent interface in a federation context openEO Platform needs to harmonise these aspects.

The data collections offered by the data centres usually all have their own names and as such appear as different data collections to a user. This might be confusing to users as they appear to be the same or very similar. Often, the data is mirrored from an official source and just converted to another file format. In this case, the data collections can be offered to the user as one collection. This means the data centres need to ensure that the names of the exposed collections (e.g., Sentinel2-L1C vs SENTINEL_2_LEVEL_1C) and the bands (e.g., B8 vs. B08) have been made consistent and that actual data values were not altered by pre-processing. Once verified, the collections can be offered as a single data collection which either means that the data offering is extended (e.g., a Europe-only archive is extended with an Asia-only archive) or the available offering is available on multiple backends so that processing can be distributed over them.

To correctly implement data harmonization, some backends with partial collections added a check for missing input data so that the availability of products can be verified upfront. This allows both the user or the Aggregator component to decide if perhaps better alternatives were available. More checks could be performed with the availability of richer and standardized STAC metadata, which is often still limited or unavailable. In the long-term, this allows backends with partial mirrors to more correctly detect cases of missing products, or even offer reprocessed products with a newer processing baseline that is not yet available at the source, all while providing full control over the product selection strategy, via standardized queries against the STAC API instances.

To allow for a consistent behaviour of the most common use cases, a set of core processes (https://docs.openeo.cloud/federation/backends/processes.html) has been agreed on. This allows parallelising several use cases across multiple backends if needed. Backends have the option to implement more specific or more complex processes, but this may limit the federation potential. The set of core processes will evolve over time and is reviewed regularly.

Workflows often end with saving the processed data to a (cloud) storage system. For this, it needs to be exported to a certain file format. The file formats and parameters themselves follow the Geospatial Data Abstraction Library (GDAL) naming convention (https://gdal.org/drivers/raster/index.html) whenever possible, but this does not necessarily mean that GDAL is required. Options that are not available through GDAL need to be harmonised across backends. Otherwise, the resulting files differ between data centres, which is not ideal if they are meant to be used together, e.g., for larger-scale processing. Some of the potential differences can be mitigated through comprehensive STAC metadata.

### Authentication and authorisation

The openEO Platform Aggregator follows the openEO API specification for authentication and authorisation. The “basic” authentication scheme, which is optional and intended for development only, is not supported as it is not feasible to operate this responsibly in a federated context. Instead, the OpenID Connect scheme is a good fit for operating in a federated context. By using OpenID Connect, the authentication and user management is isolated from the rest of the API as a separate, dedicated service. This not only eliminates security challenges and risks for individual openEO backend providers as they do not have to store or handle user credentials, it also allows using the same identity across multiple openEO backends and other services.

The OpenID Connect identity provider for openEO Platform is *EGI Check-in* (https://www.egi.eu/service/check-in/), a federated authentication service developed for the European Open Science Cloud (EOSC) and operated by the EGI Foundation. EGI Check-in acts in general as a central authentication hub, supporting multiple identity providers. A user can choose which provider they want to authenticate with: their academic institution (e.g., integrated through eduGAIN) or a social media platform (such as GitHub, Google, LinkedIn, Facebook, etc.). The first time the user authenticates through EGI Check-in, an additional registration flow is triggered to present the necessary pointers to privacy and user data handling related documents. In some cases some additional profile information might be requested.

In addition to handling authentication for end-users, the EGI Foundation also provides tools to openEO Platform to manage their user base in a so-called virtual organisation. Additional fields can be attached to users to indicate custom properties like user role, subscription level, trial expiry, etc. As noted before, this user community management is done in a central place, separate from the core business of the openEO Platform back-ends, while these can still inspect these user properties in a secure way and act accordingly.

With regards to data access, it is in the responsibility of each backend to expose data collections only to the users that have access to them. It is within the responsibility of each user to comply with the data license rules specific to the data collection (i.e., in terms of distribution and attribution). Apart from this, there is no special handling of commercial data collections in the federation. All concepts described as part of the solution (i.e., merging backend specific metadata within the aggregator, loading data from one backend into another, loading data from an external data centre into the platform) apply to both open and commercial data.

### Continental-scale processing tasks

**Fig. 4:**
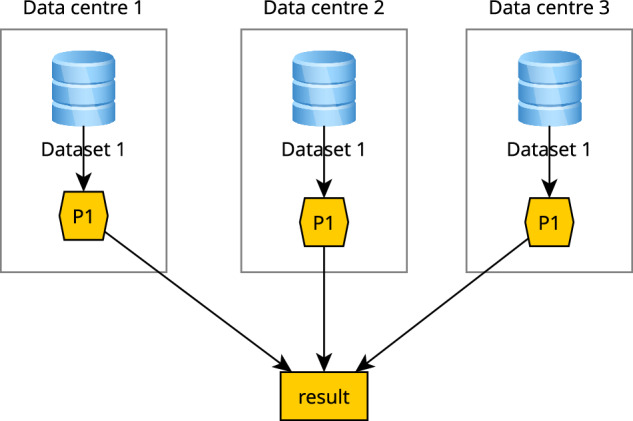
Federated processing of data: a single, continental-scale process is distributed over multiple data centres that host the same dataset and process.

For handling continental-scale processing tasks, the batch job processing mode of the openEO API is required as it can handle long-running tasks and very complex workflows. The aggregator detects the amount of data required for processing and, once a certain threshold is met or not all data is available from a single data centre, the batch job is split into smaller chunks and distributed across multiple data centres as smaller batch jobs (Fig. 4). Except for batch job API requests and metadata, this does not necessarily incur data transfer between data centres, because a central STAC catalogue that is created by the aggregator can point to the results stored in the different data centres. Due to the fact that STAC is crawlable through the links, it can point to different data centres and the STAC tooling can directly read the data from there without the user noticing it. Alternatively, a user can provision a single data centre to fetch all the results and combine them into a dataset hosted in a single premise or even into a single file.

### Accounting

Another important task involves tracking the resource utilisation across various entities within the federation. Since each platform may utilise its own methods to track system resources — such as Central Processing Unit (CPU), memory, and storage — it can become challenging to provide a unified interface. This interface should shield users from the complexity of the federation and present a coherent overview of the different resources that are consumed and those still available across the different platforms. Furthermore, users should have the flexibility to extend the available processing capacity as needed. From the platform perspective, the interface should offer a unified way to report resource usage and verify the availability of processing capacity for users.

In the context of openEO, accounting operates on a credit-based system. A *credit* is the currency of the platform, which corresponds to a fixed monetary value. This gives federation members the flexibility to use conversion rates that are appropriate for the types of resources they offer. Resource tracking is realised through a combination of internal APIs and a web application (https://openeo.vito.be/portal). The web application serves as an online interface, allowing users to manage their credits and gain insight into their credit consumption within the federation.

Metadata for batch jobs also aggregates resource usage, and the corresponding accumulated credit cost. This allows direct comparison of running the same or similar jobs with different configurations, irrespective of the backend used. If needed, all resource usage is available, allowing to identify the source of differences in total cost.

The APIs allow the different platforms to report utilisation within their respective systems. By employing a predefined set of calculation rules, the resource usage is translated into a number of credits that are deducted from the user’s account. Additionally, the APIs also allow platforms to easily check the current credit of a user. This way, platforms can prevent the execution of requested processing tasks if the user’s account lacks the necessary credits. While the technical solution to shared accounting seems fairly straightforward, it does imply that a framework is needed for handling the billing between the involved companies, which requires consideration in a federated setup.

### Aspects not covered by the federation

Building the openEO platform also revealed some aspects that could not yet be made fully transparent, often because backend providers need to be allowed a certain degree of freedom in their operational decisions.

The aspect of cost is the most important one. The backend providers are free to register costs incurred by a specific request in the central accounting system. Backends can also offer their services for free. The existing openEO federation leaves these commercial aspects to the providers, and only serves to make the service offerings comparable and interchangeable.

A caveat for the user is that the aggregator component is not able to automatically select the cheapest option yet. This would require all providers to accurately implement the openEO API functionality of providing cost estimates, which is still a technical challenge. In cases where this approach leads to user inquiries, the user is informed of alternative, cheaper options if available, allowing an immediate switch. We see this as an enormous advantage of the federation: free and commercial options operate within an open marketplace, without artificial technical or administrative burdens that slow down users in moving towards the best option for their use case.

Other aspects that are relevant for some users include the availability of cloud resources, or specific service level agreements. The federation described here does not intervene on this level, and only requires a lower bound that satisfies most use cases.

## Discussion

With the inevitable shift to cloud computing, over the last decade the Earth Observation computing landscape has fragmented, with a variety in incompatible cloud platforms, interfaces, and programming languages. Besides platform lock-in, this has led to difficulties in reproducing and validating compute platforms and resulting computations. To counter this fragmentation, we present openEO, a collection of initiatives that includes (i) an open, high-level API and a set of processes that can be used from common data science languages (R, Python, Julia, JavaScript) to process large EO datasets on a heterogeneous set of compute platforms, (ii) the ability for users to do in the cloud what they do locally, e.g., with R or Python code, and (iii) an adjacent operational public platform (openEO Platform) that allows users to carry out computations, and that handles federation of data archives or processes over various data centres (Fig. 1), in addition to billing, authentication and authorization.

openEO Platform, the operational platform that currently federates three data centres (Fig. 1) aims at integrating further data centres. A user support forum acts as a low-barrier entry point for inquiries. Technical issues and enhancement suggestions are then directed towards the GitHub issues trackers of the corresponding open source components. The ability to modify the API or add compute processes creates a good process of communication among scientists and research software engineers. The approach to create, foster and sustain a user and developer community largely aligns with the suggestions for cultivating a collaborative data science research team^14^.

openEO users can focus on what they need to do with the data rather than on how to implement it: the complexity of managing cloud infrastructure, finding files, and orchestrating the computing is hidden from users. The initiative has so far been funded by the European Commission and the European Space Agency but is open for anyone interested to participate: it is set up as a collective, community-based ecosystem of open source software components. Decisions about openEO that affect everyone undergo public discussion, and an active *project steering committee (PSC)* (https://openeo.org/psc.html) is in place.

The PSC maintains contact with related initiatives to get a mutual understanding of the commonalities and differences in approaches with the long-term goal to avoid proliferation. Two examples include the Pangeo community - for collaboration on commonly used software components such as Dask^15^ and xvec (https://xvec.readthedocs.io) - and the Open Geospatial Consortium (OGC) - for alignment with the OGC APIs. In addition, openEO is in the process of being adopted as an OGC community standard (https://portal.ogc.org/files/?artifact_id=109245version=3). This is an important step for openEO and is likely to enhance adoption in the larger geospatial domain.

For new providers, it is attractive to adopt the openEO API because of the ability to reuse the existing open-source software ecosystem, to benefit from an existing user base, and the ability for users to compare offerings from and validate results against other openEO backends. In addition, it is attractive to join the openEO Platform federation presented in this paper, in particular when offered datasets, processes, performance, and/or pricing are competitive or augment existing offerings. Joining the federation means that technical components such as authentication, authorisation, and accounting are reused, and benefits can be drawn from existing non-technical infrastructure regarding governance, training, and outreach.

The user identity management used by openEO Platform, implemented through EGI Check-in, allows for a single point of entry for users and further integration with the European Open Science Cloud. openEO and its federation concept is adopted in key Copernicus initiatives such as the new Copernicus Data Space Ecosystem. This service is responsible for the dissemination of important data sets from Sentinel-1, -2, and -3 missions, with a foreseen lifetime of at least 6 years. Having openEO integrated in this context represents a cornerstone in the shift from data download to data access and processing on cloud or high-performance computing (HPC) infrastructure.

The openEO platform federation, and the unprecedented level of harmonisation that it has achieved, also bring about new questions. For instance, when the same workflow can run in multiple locations but the costs differ: Should the aggregator spread workload evenly, or should it give preference to the cheapest provider? Should users be able to choose between cheap but slower results and fast but more expensive processing?

Using openEO to bring together large datasets from the domains of meteorology, climate modelling and Earth observations seems feasible and sensible because they all share the same time and space references and all target the wider Earth system sciences community. A federated approach would help solve the problem that these datasets are increasingly stored in different public cloud instances. Using embeddings from foundational models to compress large data cubes before transporting them is currently an area of active research. A wider integration with large datasets, e.g., from the bioinformatics or particle physics domains, seems more challenging, as the connections over space and time are less obvious, and jargon used and questions asked to experimental data have less in common with the EO or geospatial domains.

### User response

The largest user group performs relatively simple raw data access tasks. The capability to selectively extract a large variety of EO data over a very specific spatiotemporal range into a user friendly data format attracts most users. This group is the silent majority, as they rarely require support to perform these tasks.

A smaller group of users performs processing tasks like classification, integration of complex algorithms or machine learning inference via user-defined functions (UDFs, see section “Methods”). The feedback of these users indicates that there is a learning curve when it comes to building and understanding of the data cube concept, and learning the API that openEO offers. In this phase of learning, users find it frustrating that the feedback loop is slower when using a cloud based system. Using a local openEO runtime (https://openeo.org/documentation/1.0/python/client-side-processing.html) is one of the potential solutions to this particular problem. Other points of attention for this user group include making error messages as helpful as possible. The workflows created by this group sometimes also reveal functional or performance issues in the implementations.

With respect to the federation, most users eventually still end up selecting a particular backend for a given use case. Usually this happens implicitly as a result of their choice of collections and processes, or sometimes this decision is made based on aspects like cost and available processing capacity, for users with more operational requirements. In specific cases, users are unaware of the federated nature, sometimes leaving them confused, for instance when they notice a certain difference in cost.

## Methods

**Fig. 5:**
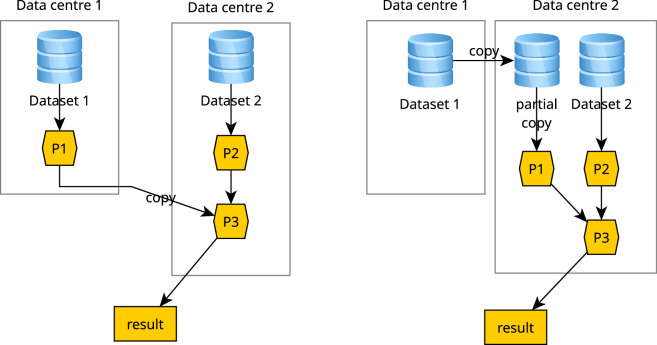
Federated processing of data. Left: processes (P1-P3) are distributed, process results are copied. Right: sub-datasets are copied, processing takes place on a single data centre.

Federated processing involves a data analysis process that needs resources (data or processing) from more than one cloud data centre. The most typical case is the combined processing of two datasets that are not available on a single data centre. The simplest processing schemes for this are shown in Fig. 5. In the left subfigure, the processing takes place on the data centres where the data resides and the results are combined in one of the data centres. In the right subfigure, the part of the dataset that is needed is copied to one data centre, where all processing takes place. Which of these strategies is optimal depends on a number of factors:

- the availability of process P1 on the data centre 1 or 2,
- the amount of data to be copied in both cases, and
- the complexity of managing processes on multiple data centres.

The classic cloud mantra “bringing the computations to the data” speaks for the left-hand solution. However, the right-hand solution may be preferable when the volume of data to be copied is relatively low. This would be the case when dataset 1 is relatively small because it has a low spatiotemporal resolution, when only a small area of dataset 1 and/or a small subset of spectral bands is required, or when only a low-resolution representation of dataset 1 is required because the user wants to compute a high-level overview over a large area without the detail of the original dataset 1 imagery. Depending on the way the imagery are organised in files in a data centre, identifying the data that needs to be copied may only involve a selection of files, but may also involve identifying, reading and copying of sections of files, which can be considered a process of its own.

When building a federated compute system, a question is to which extent users should be confronted with choices regarding how the federation takes place. A federated platform that takes over these decisions is presented in the sections before. Carrying out identical processes on different platforms assumes that processes are transferable. This becomes a larger challenge when software implementations of a process however vary between platforms, for instance in the case of machine learning or deep learning processes.

Federation and reusability concepts are not new within the EO domain. The following subsections present relevant challenges that were solved and implemented in openEO and openEO platform. They include: (i) data exchange, (ii) cloud application programming interfaces, (iii) cloud processing workflows, (iv), performance and federated capabilities by design, (v) reusability through parametrised user-defined processes, and (vi) user-defined functions.

### Data exchange

The federated processing of data as shown above opens the question of how the data centres can interact with each other. A first possible step to successfully exchange the data between the systems is to use the same file format for storage. Depending on the domain, different file formats are used. In EO, there seems to be a trend towards GeoTIFF^16^, in other domains the adoption of other file formats such as Zarr^17^ and netCDF^18^ seems to increase. Many file formats require to copy or transfer the file as a whole. This issue can be mitigated by using “cloud-native” variants of these file formats. For GeoTIFF this would be the Cloud-optimized GeoTIFF^19^, a GeoTIFF variant that allows requesting parts of the data without the need to copy the files entirely. This reduces the amount of data that needs to be transferred and with that also the time required for the transfer.

In addition to the data files themselves, it is beneficial to describe the data and enrich it with additional metadata for data search and ingestion. Metadata (de-facto) standards such as STAC allow to provide a file-format agnostic way to describe the data files. If relevant additional metadata is available in a harmonised way, it can help to exchange data between systems more easily regardless of inhomogeneous file formats. On top of the metadata, a standardised API layer such as STAC API (https://github.com/radiantearth/stac-api-spec) is useful to allow searching through the data offerings with the same language across all data centres so that the federation can look up which data is available where. This allows to identify gaps and fill them with data from another data centre. STAC (https://portal.ogc.org/files/?artifact_id=106860&version=2) and STAC API (https://portal.ogc.org/files/?artifact_id=106861&version=2) are both in the process of becoming OGC community standards. STAC API is built on top of OGC API - Features^20^.

### Cloud application programming interfaces

This subsection introduces specifications for geospatial cloud APIs and compares them. The specifications are all standardised or in the process of being standardised by the OGC. Relevant specifications are

- OGC Web Coverage Processing Service (WCPS)^21^, and
- OGC APIs with focus on OGC API - Processes^22^,
- openEO API and processes^12^.

*OGC WCPS* is a standard to formulate workflows in a language-agnostic way for *coverages*^23^. OGC WCPS is a programming language that is not extensible and not discoverable on a per-process basis, meaning that all data centres must provide the same functionality and are limited by the functionality defined in the specification.

The suite of *OGC APIs* (https://ogcapi.ogc.org) defines API layers for a variety of geospatial use cases (e.g., data discovery, data processing, and data visualisation) through *OpenAPI* (https://www.openapis.org). *OGC API - Processes* is part of it and defines the data processing part. It defines process discovery, multiple data processing modes (synchronous and asynchronous), and batch job management. It does not define actual processes that can be executed, nor does it assume a data model. This leads to implementations where workflows cannot be exchanged easily between instances. Data discovery and data visualisation are defined in other OGC API specifications, including *OGC API - Records* (https://github.com/opengeospatial/ogcapi-records) and OGC API - Tiles^24^. OGC APIs generally do not specify authentication and authorisation mechanisms.

*openEO* comprises a set of specifications and an ecosystem of open source software. The *openEO API* defines core concepts and a client-server communication API in an OpenAPI compliant way. The process specification defines a large set of processes that act on data cubes. The data processing part of the API is similar to OGC API - Processes and many other parts are defined by re-using other specifications and industry solutions. Data discovery is defined through a STAC API, whereas data visualisation can be handled through other APIs, e.g., OGC APIs - Tiles, or OGC Web Map Tile Service (WMTS). Authentication is primarily based on OpenID Connect (https://openid.net/specs/openid-connect-core-1_0.html). The technical details of the openEO API are only dealt with by software engineers who develop client interfaces or implement backend processes. End-users (data scientists) only need to use user-friendly client applications or libraries that focus on the data and processes. Clients have been developed in various languages (including R, Python, Julia and JavaScript).

From these three specifications, openEO and OGC API - Processes are most similar but have some significant differences. OGC API - Processes is a general-purpose API for data processing while the openEO API is specifically targeted towards the analysis of (virtual) data cubes on public clouds. Both the openEO API and OGC API - Processes define the HTTP API in a similar way and as such describe how the communication between clients and data centres work. This includes which endpoints exist, how they are defined schematically, and how they shall react to queries. Common examples are /processes for listing processes, /collections for listing datasets, and /jobs for job management. Both APIs can generally be extended by other OGC APIs for other purposes such as map-centric visualisations.

In terms of API specifications, components of the openEO API that are not present in OGC API - Processes include:

1. authentication retrieval of basic user details,
2. querying the available datasets through a data cube oriented interface
3. requesting the results from data processing, as specified in a process graph of arbitrary complexity, composed of openEO processes
4. specifying how the results shall be returned (specific file formats plus arbitrary additional file format options in case of download, or alternatively custom-configured OGC or non-OGC web services in case of more advanced data access or visual interaction)

At the time of the first release of openEO, OGC API - Processes and many other OGC APIs were not available or were in a very early draft stage. Due to that reason the openEO API is not fully aligned with the suite of OGC APIs yet although this is a long-term goal which needs breaking changes on either sides.

A major difference between openEO and OGC API - Processes is the availability of predefined processes. openEO defines processes for data cube oriented processing. This implicitly allows to scale up massive data volumes and allows a certain level of portability of workflows that are defined using the predefined processes.

### Cloud processing workflows

General data processing workflows can be broken down into multiple steps, often implemented as “functions” (in programming languages) or “processes” (in APIs). To federate parts of the workflows across multiple data centres it is required that each given step works in the same way. As not every data centre necessarily provides the same processing stack (e.g., Spark^25^ or Dask), these steps must be specified in a way that is agnostic to the environment and programming language. This allows a federation to run each step of the workflow independently in different data centres, if required. The *openEO processes* (https://github.com/Open-EO/openeo-processes) specification provides a common set of (EO) processes for these steps. These processes can be combined and chained into a variety of workflows. Different API specifications (e.g., OGC API - Processes, OGC WCPS, or openEO) have different ways to define workflows.

In openEO, workflows consist of a directed acyclic graph of processes and related metadata. Metadata includes a description, parameters, return values and other related information. Although openEO processes are defined as part of openEO, they are not necessarily restricted to openEO and could in principle also be combined and chained by another workflow language and executed through an API that implemented a different specification such as OGC API - Processes. Federating across different API specifications leads to more complexity in the federation though.

openEO solves some of the interoperability challenges through the openEO API specification and the process specification. A user can formulate workflows based on a predefined (but extensible) set of processes that is sent via the API to a data centre. The same workflow can also be sent to other data centres making the workflow reusable and interoperable. This results - assuming comparable datasets - in comparable results and reproducible workflows, and allows for validating data centres and/or datasets against each other.

As the processes are defined in a language agnostic way through JavaScript Object Notation (JSON), various client applications and libraries can work with them. Implementations currently exist in

- JavaScript (https://github.com/Open-EO/openeo-js-client),
- Julia (https://github.com/Open-EO/openeo-julia-client),
- Python (https://github.com/Open-EO/openeo-python-client, and
- R (https://github.com/Open-EO/openeo-r-client).

These clients enabled implementations built on top of them, such as the web-based user-interface *openEO Web Editor* (https://editor.openeo.org) based on the JavaScript client that does not require any coding to interact with openEO-compliant backends. The language-agnostic way of expressing workflows allows to convert them from one programming language into another, which enables scientists with a diverse background and knowledge of different data science languages to work together.

### Performance and federated capabilities by design

The way openEO describes data processing workflows, and the data cube oriented design of predefined processes is an enabler for achieving processing cost reduction and to allow distributed processing. The process graphs can be thought of as a declarative specification of what needs to happen, rather than a procedural sequence of fixed steps. This is comparable to the Structured Query Language (SQL) for database queries. A declarative approach leaves a lot of options for implementations to carry out advanced performance optimisations that can be tuned to the performance characteristics of the specific runtime environment.

One example technique that openEO backends implement is a so-called “push-down” of filtering processes such as slicing along data cube dimensions, but also masking at pixel level. A push-down optimisation will try to apply these filters at data load time, to reduce data loading volume, which typically drives performance in data-intensive workflows and at the same time can reduce costs in a federated set-up as less data needs to be transferred from one data centre to another.

### Reusability through parametrised user-defined processes

In openEO a process can consists of two parts: the process metadata and a processing graph. If the backend has a native implementation using their technology stack, it is a predefined process. Backends usually offer a wide variety of predefined processes that a user can combine into a user-defined process. This often consists only of a processing graph without metadata. This user-defined process can be submitted for execution at the backend. If metadata is added, in particular parameters, the parametrised user-defined process can again be re-used in other user-defined processes. This concept is comparable to programming languages that have a set of core functions (predefined processes) and pieces of business logic that the user encapsulates in functions for easy re-use (user-defined processes).

As a user-defined process is represented as a single JSON document it can be shared with other users. openEO supports parametrised user-defined processes. As such, users can create, store and share parametrised user-defined processes. This allows reusing the processes and makes processes simpler by splitting them in more manageable parts. It also allows sharing parametrised user-defined processes with other users, even across the boundaries of the backends for which they were created as long as all predefined processes are available. As such, a federated network of reusable process definitions can be established.

### User-defined functions

A user-defined function (UDF) allows users to implement certain processing steps in feature-rich languages like Python or R inside the process graphs and have them executed on the data cube. This allows ultimate flexibility for users and fills functionality gaps where the predefined processes would be lacking. It requires some trust from backends but also the availability of a runtime environment, often extended with extension packages, to be available at the backends. User-defined functions enable users to experiment with functions that are not available as predefined openEO processes, and may lead to the design and adoption of new openEO standard processes when they prove useful to a large user base.

As an example, the original Breaks for Additive Season and Trend (BFAST) method for detecting breaks in image time series^26^ was provided as an implementation in R that popularised the method. A reimplementation of the method in Google Earth Engine found an agreement in the results of only 97% (spatial) and 94% (temporal)^27^. With user-defined functions, the original implementation can be used and the challenges in porting it to a different API (such as Google Earth Engine) can be avoided.

Working with user-defined functions in backends is not trivial, both for the user and backend implementer. Some user-defined functions work only on pixel time series (like BFAST), others only on spatial areas, and others on spatiotemporal chunks of data cubes. The backends have to know these details, and adapt the chunking and parallelisation according to these needs. If the data processing is sensitive to chunk boundaries (e.g., focal operations), proper handling of overlap between chunks may be required.

For dependency management, which is needed to enable complex UDFs, the solution depends on the UDF runtime and the programming language. For instance, for Python, the language support for *inline script metadata* (https://peps.python.org/pep-0723/) allows dependencies to be declared in comments inside the UDF code, so that the backend can install them. Another emerging runtime is based on docker containers, and thus offers other dependency management features by design.

The user-defined functions require special consideration by the backend implementation in terms of security, as they allow for arbitrary code execution. Backends can choose to either only enable this feature for trusted users, much like an HPC cluster is not accessible to a wide audience, or can choose to implement typical isolation techniques to run user jobs, like many cloud environments do.

The main drawback of user-defined functions is that they can reduce some of the benefits that openEO brings in terms of open science, portability, and sometimes performance. UDFs are so powerful that users are sometimes tempted to write large parts of their processing workflow in a single UDF. It affects portability as UDFs are an advanced functionality of the openEO API, which only a small set of backends implement due to their complexity. UDFs require the availability of similar runtime environments when running workflows on multiple backends for the purpose of comparing them. The comparison of results across backends is an important aspect for open science as many cloud services are black boxes and thus scientists need to trust implementations blindly. The performance of a UDF is defined by the code and the technology stack used, and unlike workflows written with predefined processes can not be enhanced or optimised by an openEO backend.

## Data Availability

This paper does not utilise any specific dataset as it focuses on the general aspects of a federated platform.

## Code availability

All code is distributed under the Apache license, version 2.0 (https://www.apache.org/licenses/LICENSE-2.0) through the following two GitHub organisations:

• openEO (https://github.com/Open-EO/)

• openEO Platform (https://github.com/openEOPlatform)

More specifically, https://github.com/openEOPlatform/papers provides examples that show how a use case can be expressed in three different programming languages, along with the JSON process graph it will be converted into. This process can be sent to the openEO Platform aggregator and it can be distributed to and run by any of the server implementations that the aggregator is connected to. For simplicity, we skip the authentication steps in the examples. The examples show how maximum Enhanced Vegetation Index (EVI) values can be found from pixel time series of Sentinel 2 imagery.
